# An Interaction Effect of Life-Threatening Experience, Self-Efficacy, and Financial Resources on Quality of Life Among Chinese Middle-Aged and Older Women

**DOI:** 10.1007/s12126-021-09439-5

**Published:** 2021-10-11

**Authors:** Sik Yee Dion Leung, Chi Pun Ben Liu

**Affiliations:** 1 School of Health Sciences, Caritas Institute of Higher Education, Tseung Kwan O, Hong Kong; 2 Department of Social Work, Hong Kong Shue Yan University, 10 Wai Tsui Crescent Braemar Hill, North Point, Hong Kong; 3 School of Health & Social Work, University of Hertfordshire, Hertfordshire, United Kingdom

**Keywords:** Female health and well-being, Traumatic life events, Danger and opportunity, Yin Yang model of life-threatening experience

## Abstract

The current study explores the interaction effect of adversities and self-efficacy at baseline on quality of life (QoL) at follow-up among middle-aged and older Chinese women. 531 women were interviewed in 2008 and 226 of them were re-interviewed a year later using Quality of Life Ladder (QoLL), General Self-Efficacy Scale (GSE), List of Threatening Experiences (LTE), Somatic Complaint Scale, and self-rated health. Respondents’ mean age at baseline was 55.7 (SD = 4.7, range: 50–78). Over a year’s time, respondents had a decline in quality of life and self-rated health (p < .001), experienced more life-threatening events (p < .05) and somatic complaints. The hierarchical multiple regression model, employed in the study, identifies three predictors of future quality of life after adding the interaction term ‘Previous LTE × Previous GSE × Previous household income’ — previous quality of life (β = .492, p < .001), previous LTE (β = -.292, p < .001), and the interaction term (β = .221, p < .05). This model explains 34.1% of the variance of future quality of life (Adjusted R^2^ = .341, p < .001). The findings suggests that respondents’ good self-appraisal of coping resources could moderate the impact of adversities on their future quality of life. Interventions for promoting positive psychological growth among middle-aged and older adults should cover four domains, i.e. event-related factors, environmental factors, personal factors, and cognitive and coping responses. Traditional Chinese wisdom emphasizes the importance of understanding the bad (‘Yin’—the shady side) and the good (‘Yang’—the sunny side) aspect of life events. Future research may explore the Yin Yang perspective on life-threatening experiences and its applications in cross-cultural quality of life studies in the era of globalization.

## Introduction

Studies have suggested that emotional distress or post-traumatic stress disorder is ubiquitous in affected communities after disasters such as the current Covid-19 pandemic (Pfefferbaum & North, 2020). An individual's lifetime history of adversities is a strong predictor of his/her negative health and social outcomes. Adverse life events, such as ill-health, financial problems, death of a closely-related person, or unpleasant interpersonal relationships, can have negative impact on an individual’s psychological health, causing depression and anxiety (Korte et al., 2011; Li et al., 2012), an increased risk of suicide (Lau et al., 2010), substance use (Nordfjærn et al., 2010), psychiatric morbidity (Ko et al., 2001), and subjective age (Bachem et al., 2019). Childhood violence victimisation and intimate partner violence are also found to be strongly linked to such events (Yan & Karatzias, 2020). Literature has suggested that negative life events associate significantly with age-related decline in control (Cairney & Krause, 2008). The dramatic nature of adversities may also affect people’s belief in their capacity to cope with stressful situations and cause them to suffer from secondary stressors that may worsen their quality of life. However, protective factors, such as having confidence in doctors and personal precautionary measures, perceived survival likelihood, satisfaction with health information etc., may reduce the levels of stress, anxiety, and depression (Wang et al., 2020).

Quality of life, as suggested by the World Health Organization Quality of Life Group, is ‘individuals’ perception of their positions in life within the context of culture and value systems in which they live and are in relation to their goals, expectations, standards and concerns’ (Power et al., 1998:1570). There are several key predictors of quality of life, such as medical conditions and symptoms (Briancon et al., 1997), self-reported health (Yohannes & Tampubolon, 2012), having a purpose in life and self-efficacy (Urzúa et al., 2011), household income (Kivits et al., 2013), age, personality, mood, and life circumstances (Brett et al., 2012). According to Maercker and Zoellner’s Janus-Face model of self-perceived posttraumatic growth (2004), the impact of negative life circumstances on quality of life is two-sided: the constructive and self-transcending side, and the self-deceptive and illusory side. Recent research has identified three posttraumatic trajectory patterns, i.e. distressed, illusory, and constructive posttraumatic growth (Cheng et al., 2020). Yet, individuals may report illusory post-traumatic growth as a method for coping stress (Boals & Liu, 2020). Whether an individual could develop positive psychological growth after experiencing adversities, that depends on four determinants, i.e. stress-appraisal, coping styles, personality, and social support (Hildon et al., 2008; Joseph & Linley, 2006).

Studies have suggested that older persons can develop accommodative coping strategies by finding meanings from their adversities, which can enhance their adaptation and resilience to threats (Bonnano et al., 2006; de Paula Couto et al., 2011; Lager & Van Hoven, 2019; Lo et al., 2010). Resilience and self-esteem are also found to have mediated the effect of negative life events, then led to positive social adjustment (Gao et al., 2019). Older adults are more resilient and more adept at problem-solving and emotional regulation compared to younger people (Gooding et al., 2012). Frankl (1992) has argued that discovering meaning in life from negative experiences serves to transform sufferings into human achievements and accomplishments. Meaning in life can also mediate the psychological strains that influence suicide ideation (Liu et al., 2020). However, research shows that positive life changes occur only in self-efficacious people. For example, Yu et al. (2014) have found that a higher level of general self-efficacy contributed to positive posttraumatic growth among Chinese cancer survivors. Having a higher level of self-efficacy can buffer the impact of negative life events on people’s growth, quality of life, and boost their resilience to events in a stressful environment (Bandura, 1997; Prati et al., 2010). Therefore, interventions immediately after adverse life events may reduce the risk of cognitive decline among older adults (Tian et al., 2020).

Self-efficacy is how people perceive their competence and abilities in coping with life's challenges. It influences the willingness, effort, and perseverance of an individual may put forth in the face of adversities, which is therefore a personal resource factor positively predicting problem-focused coping (Bandura, 1997; Troillet et al., 2011). Also, it is an agentic factor in posttraumatic recovery and other psychiatric disorders (Hoelterhoff & Chung, 2013). For example, a meta-analysis has consolidated results of studies and then found coping-specific self-efficacy has a strong association with lower levels of posttraumatic stress disorder symptoms (Gallagher et al., 2020). However, Cairney and Krause (2008) has argued that existing literature overlooks older adults’ exposure to negative life events which are the important predictor of age-related decline. It has also been hypothesized that Chinese people’s cognitive adaptation to stress is not identical with that of the western people (Chan et al., 2006), because Chinese people may adopt a different approach to adapt to their environment (Yu & Zhang, 2007). There are also limited longitudinal studies exploring the interaction effect of life-threatening experiences, self-efficacy, and financial resources on the quality of life among older Chinese women. In view of that, the current study aims to fill the gap and tests the following null hypothesizes:

1. The single factor of previous life-threatening experiences, self-efficacy, and financial resources alone does not establish a statistically significant relationship with a better future quality of life among middle-aged and older Chinese women
2. The interaction effect of previous life-threatening experiences, self-efficacy, and financial resources does not establish a statistically significant relationship with a better future quality of life among middle-aged and older Chinese women

## Methods

### Setting

The study was based on a lifelong learning program for women developed and subsidized by Women’s Commission of Hong Kong Special Administrative Region Government (HKSAR) (Women’s Commission, 2019). It was launched in March 2004, providing female adult learners with unconventional, good-quality and specially designed non-award bearing courses delivered via radio broadcasts as well as by face-to-face teaching modes (HKSAR, 2004; WoD, 2003). Courses offered in the Program included arts and culture, interpersonal relationships and communication skills, finance management, health, and other practical issues relating to daily living (HWFB, 2005; Women’s Commission, 2019). Up to January 2020, the cumulated enrolment in this program was over 110,000 (Women’s Commission, 2020).

### Participants and Recruitment

The target group of the current study consisted of middle-aged and older female learners. There was a total of 6,500 students enrolled in 2008 (HKSAR, 2009) and all of them were sent a letter in March 2008, inviting them to participate in this study. Instead of selecting respondents via a sampling method, the current study used the entire population of the program. A total of 1,003 students responded to our invitation and 531 respondents fulfilled the inclusion criteria for this study, i.e. aged 50 years or older. A follow up was made in August 2009 and 226 of the respondents were re-interviewed.

### Data Collection Procedures

All participants were sent a self-administered questionnaire, which was formulated in English and then translated into Chinese. The Chinese version was back-translated to English again to examine whether the contents were well-translated. The questionnaire used for data collection was the Chinese version, and these Chinese-translated measurement tools have been tested in a pilot study. Thirteen adult learners filled the Chinese-translated questionnaire to test the inter-correlation among items of the questionnaire. Results of the pilot study was satisfactory (Cronbach’s alpha was 0.941 for General self-efficacy (GSE) scale, 0.512 for List of threatening experiences (LTE), and 0.857 for Somatic complaint scale (SCS)). As the LTE is just a checklist for calculating the number of critical life events rather than a measurement scale, the low Cronbach’s score did not affect its reliability. Details of the measurements are as follows:

A) Dependent variable
  The Quality of Life Ladder (QoLL), which is a single item leader-shaped visual analogue scale, was adopted to assess a participant’s level of quality of life level. The score anchors from ‘(0) worst possible’ to ‘(10) best possible’, indicating that the higher the score, the better the quality of life (Cantril, 1965). Bowling (2005) argued that a single item measure had the obvious benefit of an easy interpretation of a respondent’s subjective health status.
B) Independent variables

1. The List of Threatening Experiences (LTE) is a modified 6-item inventory of life events by reporting the number of threats that respondents have experienced in the last 12 months (Brugha, et al., 1985). A higher LTE number indicates having more adverse life events (ranging from 0 to 6). Face validity of the inventory was good (Brugha, et al., 1985) though the Cronbach’s alpha for the Chinese version is 0.554.
2. General Self-Efficacy Scale (GSE) (Zhang & Schwarzer, 1995) is a 10-item tool to examine respondents’ personal competence in dealing efficiently with a variety of stressful situations, with higher scores indicating a better self-efficacy (ranging from 10 to 40). The Cronbach’s alpha for the Chinese version is 0.906.
3. Somatic Complaint Scale (SCS) (Scott & Lindberg, 2000) is a 12-item scale measuring a respondent’s degree of discomfort experienced from somatic complaints, with higher scores indicating having more health problems (ranging from 0 to 12). The Cronbach’s alpha for the Chinese version is 0.879.
4. Self-rated health (SRH): a single-item measure asking respondents to rate their health as ‘5 = Very Good’, ‘4 = Good’, ‘3 = Fair’, ‘2 = Poor’, or ‘1 = Very Poor’.
5. Sociodemographic variables include age and monthly household income.

### Data Management and Analysis

Using IBM SPSS version 25, the changes of score in measurement tools over time were examined by t-test, ANOVA, and Chi-square test. Hierarchical multiple regression analysis was applied to explore how independent variables and covariates at baseline predicted quality of life at follow-up. The regression model also examined the interaction effect of financial resources, self-efficacy, and life-threatening experiences at baseline on quality of life at follow-up.

### Ethics Statement

The research protocols were approved by the ethics approval committee of the University where the study was conducted (Project No.: 07/1.12). Informed consent was sought from all respondents who participated in this study.

## Result

### Respondents’ Characteristics

A total of 531 middle-aged and older Chinese female learners participated in the baseline survey in 2008 and 42.7% (n = 226) of them returned their questionnaires at follow-up in 2009. Though the attrition for follow-up was high, there were no statistical differences in the key measures including LTE, GSE, SCS, and SRH between the remaining group and the drop-out group. Despite a relatively lower QoL mean score in the drop-out group compared with the remaining group (*t* = 2.733, df = 516, p < 0.01), the result of Little’s Missing Completely at Random (MCAR) test (Little, 1988) shows that our data are MCAR and no patterns exist in the missing data (χ^2^_(df 5)_ = 5.073, p = 0.407). In other words, the missingness is completely unsystematic and unrelated to the study variables.

Table 1 shows that the mean age of the remaining group at baseline is 55.7 (SD = 4.7, range: 50–78). Slightly more than one-fifth of the respondents had full-time employment and the average monthly household income dropped to US$2,821 from US$3,161 over a year’s time. The mean score of quality of life decreased from 6.54 to 6.36 (p < 0.001); about one-tenth of respondents perceived their health as poor or very poor (p < 0.001), and most of them suffered at least one somatic complaint. Slightly more than a half of respondents experienced at least one LTE at baseline and the prevalence was increased to 54% from 52.8% over time (p < 0.05). There was a 41.3% increase in relation to something valuable being lost or stolen (p < 0.001) and a 38.7% increase in major financial crisis (p < 0.001) over time. Those respondents who did not have LTE at baseline but experienced at least one LTE at follow-up perceived a better quality of life at follow-up than their counterparts (Table 2). When examining the effects of LTE and GSE at baseline on quality of life at follow-up, Table 3 reports that about two-thirds of those respondents who had at least one LTE and a higher GSE at baseline perceived a better quality of life at follow-up (p < 0.05, Table 3). Respondents who had a higher monthly household income achieved a better GSE and quality of life compared to those with a lower household income (Table 4).

**Table 1:** Changes of scores over time

	Baseline (N = 531)	Follow-up (N = 226)
Age	55.7 (SD = 4.7)	55.6 (SD = 4.3)
Had full-time employment	119 (22.4%)	53 (23.8%)
Average monthly household income	US$3161 (SD = US$2221)	US$2821 (SD = US$3162)
Quality of Life Ladder	6.54 (SD = 1.5, range 1–10)	6.36 (SD = 1.4, range 2–10) ^1^
Self-rated health Very poor Poor Fair Good Very Good	3 (0.6%) 37 (7.0%) 316 (59.6%) 159 (30%) 15 (2.8%)	3 (1.3%) 20 (8.9%) 121 (54%) 74 (33%) 6 (2.7%) ^2^
At least one somatic complaint	514 (97%)	221 (99%)
At least one life-threatening event	280 (52.8%)	122 (54%) ^3^
General Self-Efficacy Scale	26.06 (SD = 5.8, range 12–40)	25.49 (SD = 5.7, range 10–40)

All are not significant except

^1^*t*_(df 223)_ = 4.253, *p* < .001

^2^χ^2^_(df 12)_ = 148.281, *p* < .001

^3^*t*_(df 224)_ = -1.995, *p* < .05

**Table 2:** Changes of life-threatening events and key measures over time

Variables at follow-up	Changes of LTE over time^1^				
	No – No	Yes – Yes	No – Yes	Yes – No	Total
Quality of Life Ladder ^2^	6.70 (SD = 1.1)	6.00 (SD = 1.6)	6.78 (SD = 1.6)	5.94 (SD = 1.4)	6.35 (SD = 1.5)
General Self-Efficacy Scale ^3^	25.13 (SD = 5.5)	26.28 (SD = 5.8)	25.48 (SD = 6.7)	24.03 (SD = 4.2)	25.45 (SD = 5.7)
Somatic Complaint Scale ^4^	7.57 (SD = 2.9)	8.65 (SD = 3.1)	8.09 (SD = 3.1)	8.96 (SD = 2.8)	8.25 (SD = 3.1)
Self-rated health ^5^	3.39 (SD = 0.6)	3.10 (SD = 0.7)	3.29 (SD = 0.7)	3.38 (SD = 0.6)	3.26 (SD = 0.7)

^1^*No–No* Baseline and follow-up  0, *Yes–Yes* Baseline and follow-up > 0, *No–Yes* Baseline  0 Follow-up > 0, *Yes–No* Baseline > 0, Follow-up = 0

^2^F_(df=3;219)_ = 5.011, p < .01

^3^F_(df=3;220)_ = 1.323. p = n.s

^4^F_(df=3;218)_ = 2.280; p = n.s

^5^F_(df=3;219)_ = 2.405, p = n.s

**Table 3:** Life-threatening events (LTE), self-efficacy (GSE), and quality of life ladder (QoL)

LTE at baseline	GSE at baseline	Low QoL at follow-up ^1^	High QoL at follow-up ^1^	Total (%)
> = 1	Lower than & equal to the mean	43 (62.3)	18 (41.9)	61 (54.5)
	Higher than the mean	26 (37.7)	25 (58.1)	51 (45.5)
	Total	69 (100)	43 (100)	112 (100) ^2^
= 0	Lower than & equal to the mean	26 (56.5)	31 (47.0)	57 (50.9)
	Higher than the mean	20 (43.5)	35 (53.0)	55 (49.1)
	Total	46 (100)	66 (100)	112 (100) ^3^

^1^*Low QoL* Lower than and equal to the mean, *High QoL*  higher than the mean

^2^_(df 1)_ = 4.471, *p* < 0.05

^3^χ^2^_(df 1)_ = 0.990, *p* = .211

**Table 4:** Financial conditions, self-efficacy (GSE), and quality of life ladder (QoL)

Monthly Household income at baseline	GSE at baseline	QoL at baseline	QoL at follow-up
Below US$2500	25.52 (5.7)	6.15 (1.5)	6.22 (1.5)
US$2501 – US$3800	25.40 (5.6)	6.59 (1.4)	6.12 (1.5)
US$3801 and over	27.72 (5.8)	7.21 (1.4)	6.77 (1.4)
Total	26.06 (5.7)	6.56 (1.5)	6.32 (1.5)
ANOVA	F_(2,459)_ = 6.919, p < .001	F_(2,460)_ = 19.421, p < .001	F_(2,203)_ = 3.140, p < .05

From the results of hierarchical multiple regression analysis (Block 1, Table 5), the only significant predictor of quality of life at follow-up was the level of quality of life at baseline (Adjusted R^2^ = 0.309, p < 0.001). However, after adding LTE and GSE at baseline in the equation (Block 2), the quality of life and LTE at baseline were found to be strong predictors of future quality of life (Adjusted R^2^ = 0.324, ΔR^2^ = 0.025, p < 0.001). The LTE factor can explain an additional 2.5% of variance of future quality of life. After adding the interaction term ‘LTE at baseline × GSE at baseline × Household income at baseline’ in Block 3, three key predictors of future quality of life were found: previous quality of life (β = 0.492, p < 0.001), previous LTE (β = -0.292, p < 0.001), and the moderation effect of ‘LTE at baseline × GSE at baseline × Household income at baseline’ (β = 0.221, p < 0.05). The model can explain 34.1% of the variance of future quality of life (Adjusted R^2^ = 0.341, ΔR^2^ = 0.020, p < 0.001).

**Table 5:** Hierarchical regression results of predictors of quality of life ladder^1^ at follow-up

Variables at baseline	B (std error)	β	P-value
*Block 1*			
Quality of life ladder score^1^	.591 (.067)	.556	.000
Somatic Complaint Scale score^2^	.002 (.034)	.003	.958
Level of self-rated health^3^	.065 (.172)	.025	.705
F _(3,202)=_31.541, p < .001, Adjusted R^2^ = .309			
*Block 2*			
Quality of life ladder score	.534 (.072)	.502	.000
Somatic Complaint Scale score	.007 (.034)	.013	.840
Level of self-rated health	.062 (.170)	.024	.713
Number of life-threatening events (LTE)^4^	-.229 (.087)	-.159	.009
General Self-Efficacy Scale score (GSE)^5^	.171 (.175)	.058	.331
Monthly household income	.007 (.110)	.004	.946
F _(6,199)=_17.367, p < .001, Adjusted R^2^ = .324, Δ R^2^ = .025			
*Block 3*			
Quality of life ladder score	.524 (.071)	.492	.000
Somatic Complaint Scale score	.011 (.034)	.021	.749
Level of self-rated health	.115 (.169)	.045	.496
Number of life-threatening events (LTE)	-.421 (.115)	-.292	.000
General Self-Efficacy Scale (GSE) score	-.103 (.205)	-.035	.617
Monthly household income	-.084 (.115)	-.045	.465
Interaction: LTE x GSE x Monthly household income	.193 (.078)	.221	.014
F _(7,198)=_16.144, p < .001, Adjusted R^2^ = .341, Δ R^2^ = .020			

^1^A higher score indicates a better the quality of life (ranging from 0 to 10)

^2^A higher score indicates having more health problems (ranging from 0 to 12)

^3^5 Very Good, 4 Good, 3  Fair, 2 Poor and 1 Very Poor

^4^A higher number indicates suffering more adverse life events (ranging from 0 to 6)

^5^ A higher score indicates a better self-efficacy (ranging from 10 to 40)

## Discussion

As positive expectancies, such as self-efficacy, optimism, hope, are associated with lower levels of posttraumatic stress symptoms (Gallagher et al., 2020), self-efficacious people may perceive life-threatening events as controllable and meaningful. However, the current study found a declining trend in self-efficacy among middle-aged and older Chinese women over time and self-efficacy alone was found not a significant predictor of the future quality of life. Studies have suggested that trauma survivors may feel helpless in life-threatening events, which may diminish their self-efficacy beliefs (Morina et al., 2018). Individuals may develop confidence based on flawed judgements, inaccurate memories or erroneous opinions and peer models; and thus, self-efficacy alone cannot guarantee effective results (Petrovich, 2004). Furthermore, self-efficacy may have a weaker effect on goal progress when goal importance is low and in a multiple-goal environment with limited resources (Beattie et al., 2015). In other words, the influence of self-efficacy alone on coping with trauma may be limited.

Research has reported that financial stress is a trigger for the development of depressive symptoms among older Chinese women (Lin et al., 2011), and financial constraints significantly predict depression among middle-aged and older adults (Gillen et al., 2017). Unlike previous studies, the current study has found that household income alone was not a significant predictor of future quality of life. However, the combination of coping resources, i.e. combing personal resources, such as self-efficacy beliefs and that of social resources such as financial assistance, is documented as a protective factor of well-being among older people. For example, social support is a direct agent providing emotional, instrumental, and informational support to increase an individual’s psychological empowerment and mental health (Liu et al., 2017). The current study confirms the combined effect of personal and social resources, reporting that respondents from a better-resourced family with a good self-efficacy and experience of life-threatening events perceived a better future quality of life. Therefore, from the findings of our current study, the interaction effect of financial resources, self-efficacy beliefs, and life-threatening events on future quality of life has been confirmed. In other words, our respondents’ self-efficacy beliefs, which were enhanced by the sufficient financial resources, successfully transformed the negative life challenges into positive psychological growth and well-being.

Literature reveals that greater availability of coping resources can facilitate better well-being in older adults (Boehlen et al., 2017; Jonker et al., 2009). Coping resources is normally assumed to be a consistent moderator of adversities on psychological well-being because, according to the stress-suppressing model, sufficient resources can reduce psychological distress. However, the moderating effect of coping resources depends on the scope and intensity of the stressor. Stress-conditioning model therefore argues that the stressor moderates the pre-existing resources in the prediction of stress, indicating that a high-intensity stressor may induce distress when coping resources are insufficient (Ensel & Lin, 1991). The current study has found no evidence to support the stress-suppressing model or stress-conditioning because these models do not consider the role of self-efficacy beliefs in the interaction process. On the contrary, our findings echo the argument of Prati et al. (2010) that a good self-appraisal of coping resources can moderate the impact of stressors on quality of life. Older adults can still recover from the stressors because adverse life events may not generate pervasive and long-term negative effects on life (Cairney & Krause, 2008).

Our findings support Janus-Face model's self-perceived posttraumatic growth (Maercker & Zoellner, 2004), suggesting that Chinese seniors in the current study could convert crises into opportunities for self-transcendence. According to Lu and Hsieh (2013), Chinese culture has a strong influence on older adults’ perception of negative life challenges. Older adults are also more efficient in emotional recovery after suffering from adversities compared to younger adults (Pearman et al., 2010) because they are having more time to reflect on their critical life experiences which they can consolidate into a meaningful sense of spiritual life (Lo et al., 2010). Then, the meaning-making on stressful conditions can become a powerful accommodative coping strategy for adaptability and resiliency (Turner et al., 2012). On the other hand, those Chinese seniors who had fewer financial resources may develop a dysfunctional and self-deceptive belief of their abilities to succeed in adversities. Even though such self-perception may counterbalance emotional distress (Zoellner & Maercker, 2006), it could contribute negatively to quality of life.

## Practical Implications

The current study has identified an important interaction effect of previous life-threatening events, self-efficacy, and financial resources on the future quality of life among Chinese female seniors. Although adverse life events are irreversible, there are modifiable factors, such as financial support and self-efficacy beliefs, that have the potential to facilitate the development of positive psychological growth and wellbeing in older adults. Literature shows several determinants of posttraumatic growth including event centrality, environmental factors, emotional regulation personal factors, cognitive, religious, or spiritual coping, and posttraumatic stress, (Schaefer & Moos, 1998; Xia, 2017). Event centrality that is recurrent event-related positive thinking, also known as rumination, makes sense of life challenging experiences, and resolve the problems generated from the incident (Hallam & Morris, 2014). Middle-aged and older adults can re-establish core beliefs and assumptions about themselves and the world through deliberate rumination, which is demonstrated to be a direct cause of posttraumatic growth (Taku & Oshio, 2015). Additionally, ‘approach coping’ that includes positive cognitive reappraisal and behavioural coping strategies can eliminate illusory coping responses and avoidance coping through actively seeking guidance and support for problem-solving. However, Schuettler and Boals (2011) found that resilient and self-efficacious individuals may not experience growth if they do not perceive the event as traumatic or stressful. Therefore, they can be provided culturally relevant and growth-focused training to enable them to discover meanings and positive perceptions of negative life events. For example, the strength-focused and meaning-oriented approach for resilience and transformation model was found useful in helping middle-aged Chinese survivors of severe acute respiratory syndrome (SARS) (Ng et al., 2006). The group-based culturally adapted interventions have also been reported effective in tackling mental health issues during Covid-19 pandemic (Yue et al., 2020). Environmental factors, including the provision of social resources and social supports, can facilitate middle-aged and older adults to build up buffers against adversities. Social support has been well-reported in the literature to be positively associated with posttraumatic growth.

Maercker and Zoellner’s (2004) Janus-Face model of posttraumatic growth suggests that life circumstances can be both positive and negative to the quality of life, depending on the individuals’ self-appraisal of coping capacities. Though the current study has not applied measurement tools to explore the role of culture on Chinese seniors’ perceptions towards adversities, we can still make reference to Chinese cultural wisdom to understand the life-threatening experiences among middle-aged and older Chinese women. The word ‘crisis’ in Chinese comprises two elements: danger (*wēi* 危*)* and opportunity (*jī 機*) (Zimmer, 2007). For example, the recent Covid-19 pandemic presents both a danger and an opportunity: a hidden gift and a devastating loss (Yang, 2020). The meaning of danger and opportunity (*wēi jī* 危機) is identical with the Western concept of crisis, and that is to tell a latent danger (Mair, 2009). On the other hand, *jī* (Opportunity) indicates a critical moment for better or worse, and that is like the meaning of ‘crisis’ in English — ‘the moment in which a decision was about to occur’ (Graf, 2010: 599). The concept of crisis combines the notion of objective crisis and subject critique, i.e. the diagnostic and prognostic elements. Simply put, the manner people conceptualize the critical stage in their lives predicts what will happen to their lives in the future. A crisis in Chinese culture represents a decisive moment for a critical change in life. Whether an individual can achieve personal growth and self-transcendence depends on his/her self-appraisal of coping capacities and personal resources to manage stressful life events. Adversity is a determinant for better self-efficacy (Solberg et al., 2005) because people can re-organize themselves and their lives to cope with stress and challenges (Turner et al., 2012). This indicates that human beings have the potential to transform adversities into positive growth in their lives. A study on the impact of severe acute respiratory syndrome on older Chinese adults found that Chinese seniors demonstrate high psychological resilience in coping with the unpredictable health threat (Lau et al., 2008). That shows individuals’ perceived capacity to manage their personal functioning in handling stressful life events can generate positive recovery in their social, cognitive, emotional, physical and spiritual / philosophical aspects of life, such as improving relationship with others and having a changed sense of self after critical life events (Benight & Bandura, 2004; Tedeschi & Calhoun, 2004). The Yin Yang theory is a traditional Chinese duality thinking (Hung, 2020). The concept of Yin (陰the shady side) and Yang (陽the sunny side) indicates that though different people may interpret the word ‘crisis’ differently, they need to strike a balance between the two opposite dynamics (Jiang, 2013), and by adopting this perspective people can identify a balanced approach for self-transience in challenging environments. Wrisberg and Fisher (2005) has applied the Yin Yan concept to injury rehabilitation and concluded that this traditional Chinese wisdom emphasizes the importance of understanding the good and bad aspects of life events. Future research may explore the Chinese Yin Yang perspective further on life-threatening experiences and its applications in cross-cultural quality of life study in the era of globalization.

## Limitations of the Study

There are several limitations to this study. Firstly, respondents of the current panel study were drawn from a large-scale lifelong educational program, instead of the whole population of Hong Kong. This may generate a biased result as active learners tend to perceive their psychological well-being and self-efficacy more positively. Even so, because the program is a government-funded territory-wide community project, which has a wide coverage in Hong Kong (HKSAR, 2009). The current study therefore could fill a gap in our existing knowledge by providing an insight into how middle-aged and older Chinese adults respond to critical life events. Secondly, the samples were not randomly selected, and the response rate was low. Our respondents were active adult learners who were relatively motivated and optimistic about aging and life. To a certain extent, this limits the generalization of the study so caution should be taken in interpreting our data. However, we have compared all the key measurements between the remaining respondents and the dropouts by conducting Little’s Missing Completely at Random test. Doing so has confirmed that the missing data does not result in any bias in this study. Thirdly, instead of using face-to-face interviews directly assessing the psychological health of our respondents, the current study has adopted self-report measures of quality of life and other psychosocial aspects. Yet, it has been documented that self-reported measures are commonly accepted as a valid assessment of psychological health. Despite these limitations, the current study has provided empirical longitudinal data on the impact of adverse life events on quality of life among Chinese seniors. It has also provided a cross-cultural perspective for understanding the interaction effect of life-threatening experiences, self-efficacy, and financial resources on quality of life among middle-aged and older adults.
